# Observation of morphological abnormalities in silkworm pupae after feeding ^137^CsCl-supplemented diet to evaluate the effects of low dose-rate exposure

**DOI:** 10.1038/s41598-020-72882-y

**Published:** 2020-09-29

**Authors:** Sota Tanaka, Tadatoshi Kinouchi, Tsuguru Fujii, Tetsuji Imanaka, Tomoyuki Takahashi, Satoshi Fukutani, Daisuke Maki, Akihiro Nohtomi, Sentaro Takahashi

**Affiliations:** 1 Research Group for Environmental Science, Japan Atomic Energy Agency, Tokai, Ibaraki 319-1195 Japan; 2 Division of Radiation Life Science, Institute for Integrated Radiation and Nuclear Science, Kyoto University, Kumatori-cho, Sennan-gun, Osaka, 590-0494 Japan; 3 Laboratory of Creative Science for Insect Industries, Graduate School of Bioresource and Bioenvironmental Sciences, Kyushu University, Nishi-ku, Motooka, Fukuoka, 819-0395 Japan; 4 Division of Nuclear Engineering Science, Institute for Integrated Radiation and Nuclear Science, Kyoto University, Kumatori-cho, Sennan-gun, Osaka, 590-0494 Japan; 5 Technical Staff Office, Institute for Integrated Radiation and Nuclear Science, Kyoto University, Kumatori-cho, Sennan-gun, Osaka, 590-0494 Japan; 6 Quantum Radiation Sciences, Department of Health Sciences, Graduate School of Medical Sciences, Kyushu University, Maidashi, Higashi-ku, Fukuoka City, 812-8582 Japan; 7 Professor Emeritus, Kyoto University, Kitashirakawa Oiwake-cho, Sakyo-ku, Kyoto, 606-8502 Japan

**Keywords:** Environmental sciences, Environmental impact

## Abstract

Since the Fukushima Dai-ichi Nuclear Power Plant (FDNPP) accident, morphological abnormalities in lepidopteran insects, such as shrinkage and/or aberration of wings, have been reported. Butterflies experimentally exposed to radiocesium also show such abnormalities. However, because of a lack of data on absorbed dose and dose–effect relationship, it is unclear whether these abnormalities are caused directly by radiation. We conducted a low dose-rate exposure experiment in silkworms reared from egg to fully developed larvae on a ^137^CsCl-supplemented artificial diet and estimated the absorbed dose to evaluate morphological abnormalities in pupal wings. We used ^137^CsCl at 1.3 × 10^3^ Bq/g fresh weight to simulate ^137^Cs contamination around the FDNPP. Absorbed doses were estimated using a glass rod dosimeter and Monte Carlo particle transport simulation code PHITS. Average external absorbed doses were approximately 0.24 (on diet) and 0.016 mGy/day (near diet); the average internal absorbed dose was approximately 0.82 mGy/day. Pupal wing structure is sensitive to radiation exposure. However, no significant differences were observed in the wing-to-whole body ratio of pupae between the ^137^CsCl-exposure and control groups. These results suggest that silkworms are insensitive to low dose-rate exposure due to chronic ingestion of high ^137^Cs at a high concentration.

## Introduction

The Tokyo Electric Power Company’s Fukushima Daiichi Nuclear Power Plant (FDNPP) accident in March 2011 caused severe radioactive contamination across a wide area in eastern Japan. If the radiation dose is sufficient to induce radiation hazards, it is necessary to take appropriate measures for the radiological protection of non-human biota^1^. Several studies have been conducted to evaluate the effects of radiation on wildlife after the accident. Field studies have shown chromosomal aberrations and enhanced spermatogenesis in relatively high radiosensitive field mice^2–4^. Furthermore, radiation effects on hematopoiesis have been demonstrated in wild Japanese monkeys^5,6^. Morphological abnormalities have been observed in conifer trees^7,8^, gall-forming aphids^9^, and butterflies^10–13^. Of note, lepidopteran insects have holocentric chromosomes, and they are highly tolerant of radiation-induced chromosomal aberrations^14–16^. The International Database on Insect Disinfestation and Sterilization (IDIDAS)^17^, containing more than 2750 references, provides information on radiation doses that induce mortality and reproductive sterilization in arthropods for pest control. The data and indicate that the lepidopteran insects (Arctiidae, Pyralidae) are the most radioresistant among all arthropods reported in of the database.

Nonetheless, morphological abnormalities in lepidopteran butterflies have been reported after the FDNPP accident^10–13^. A decrease in the abundance of butterflies has also been reported after the Chernobyl and Fukushima nuclear accidents^18,19^. To verify whether these observed effects on wild species are caused by radioactive contamination, accurate estimation of radiation dose and assessment of the dose–effect relationship is necessary. However, to the best of our knowledge, such data for insects have not been presented^9–13,18,19^.

Data from field studies under contaminated conditions after the accident are necessary to evaluate the environmental effects of radiation. However, it is difficult to accurately evaluate the radiation effects from the data of environmental analysis because field data are limited by several uncertainty factors such as insufficient absolute number of samples, nonuniformity of environmental conditions, and biological differences among tested individuals. To overcome these limitations, a laboratory approach is required primarily to test the effects of low doses and low dose-rate exposure. However, experimental data are insufficient to verify the effects of low dose and low dose-rate exposure in insects^1^.

Some internal exposure experiments have been conducted by feeding radiocesium-absorbed leaves to the pale grass blue butterfly, *Zizeeria maha*^20–22^. These experiments have showed high mortality and morphological abnormalities in the butterfly at low levels of exposure. Taira et al.^23^ also conducted an internal exposure experiment in the cabbage white butterfly, *Pieris rapae*, using radiocesium-contaminated leaves and observed developmental and morphological abnormalities. These results suggest that the lepidopterans are highly sensitive to radiocesium internal exposure; thus, accurate absorbed dose estimation is required. However, there are no laboratory studies on such dose estimation.

Silkworm has been used in studies on radiation biology, radiation genetics, and radiation entomology, resulting in the accumulation of data on radiation effects^24–29^. These data are essential to understand the consequences of radiation in silkworms. However, these data have been obtained mainly by high-dose external irradiation and, thus, it is difficult to adapt the data to evaluate low-dose radiation effects.

**Figure 1:**
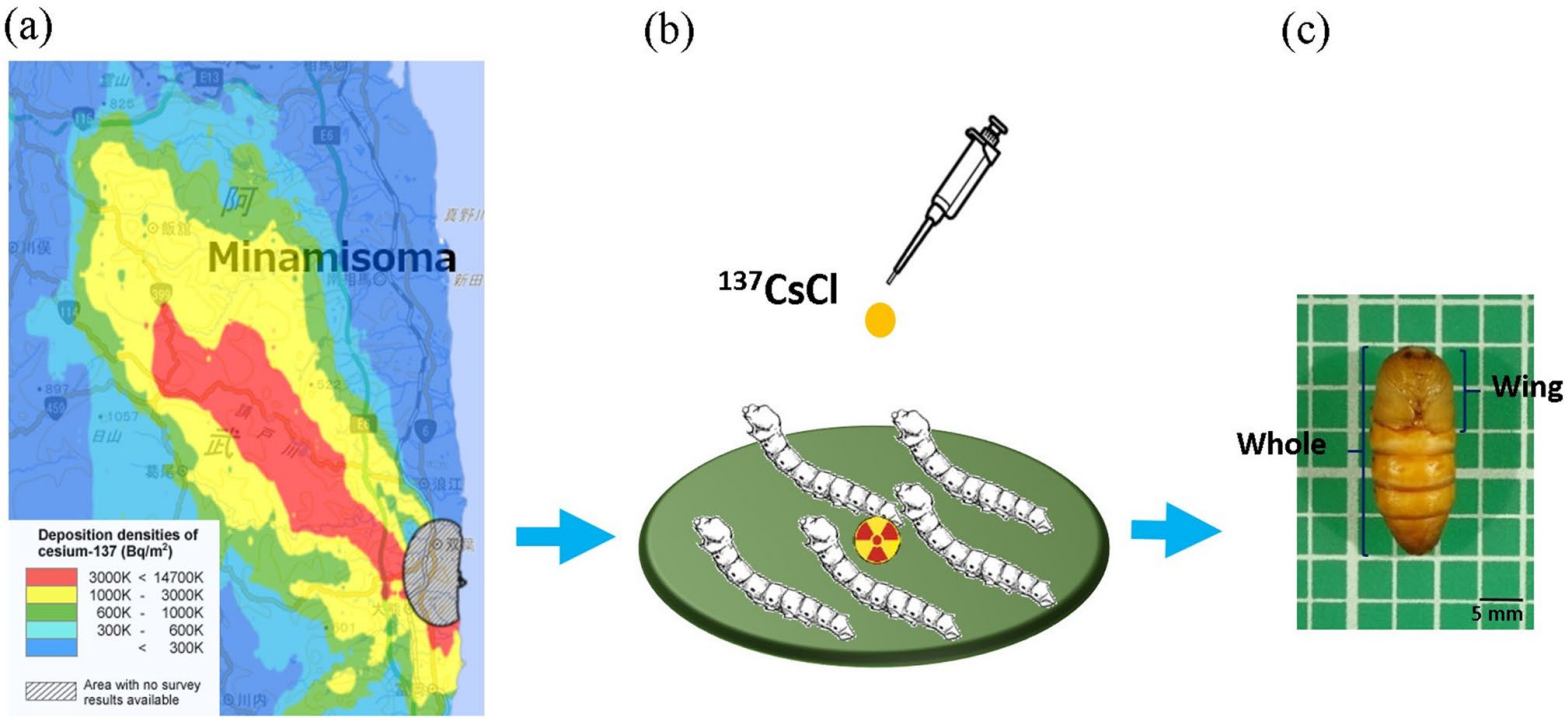
Internal exposure experimental system of silkworm. (**a**) Initial deposition density of ^137^Cs after the FDNPP accident, airborne survey on April 29, 2011. The map was generated using ‘Extension Site of Distribution Map of Radiation Dose, etc.,/GIS Maps’^40^. (**b**) Silkworms were reared on ^137^CsCl-supplemented artificial diet set to the activity concentrations of ^137^Cs in the red area in the map. (**c**) Measurement of the wing-to-whole body length ratio of silkworm pupae as an indicator of morphological abnormalities caused by radiation.

In this study, we used the silkworm, *Bombyx mori*, as a model lepidopteran to evaluate the relationship between the absorbed dose of radiocesium and morphological abnormalities in pupal wings at low-dose exposure (Fig. 1). Our results are relevant considering a lack of dose estimation studies in insects. They will prompt future studies to evaluate the effects of chronic low dose-rate radiation exposures in lepidopteran insects.

## Results

### External absorbed dose rate

The external absorbed dose rate estimated using a glass rod dosimeter is summarized in Table 1. The absorbed dose rate on the diet was 0.24 mGy/day and the ratio of beta to gamma rays was 0.55. The absorbed dose-rate near the diet (20 mm from the edge of the diet pellet) was 0.016 mGy/day and the ratio of beta to gamma rays was 0.14. When the silkworms were assumed to live on the diet for 29 days and near the diet for 3 days, the total external absorbed dose by the silkworm was 6.9 mGy. The average external absorbed dose rate of the silkworm was 0.21 mGy/day.

**Table 1:** Absorbed dose rate determined by glass rod dosimeter.

mGy/day	γ-ray	β-ray	γ + β	β/γ
On the diet	0.15	0.084	0.24	0.55
Near the diet	0.014	0.0020	0.016	0.14

### Internal dose rate

The absorbed dose-rate upon internal exposure was estimated using the Particle and Heavy Ion Transport code System (PHITS). The mean weight, length, width, ^137^Cs concentration, and ^137^Cs activity of the fifth instar larvae are summarised in Table 2. The absorbed dose rate of electrons and photons was 0.79 and 0.027 mGy/day, respectively (Table 3). The internal exposure period, which is the total larval stage period, was 20 days, and the total internal dose was approximately 16 mGy. The average internal absorbed dose-rate of silkworm was 0.82 mGy/day.

**Table 2:** Mean weight and concentration of ^137^Cs in the fifth instar larvae.

Weight (g)	Length (cm)	Width (cm)	^137^Cs (Bq/g fw)	Activity (Bq)
1.72 ± 0.18	4.38 ± 0.16	0.76 ± 0.08	274 ± 33.5	470 ± 79.9

± Standard deviation.

**Table 3:** Internal dose rate estimated using PHITS.

	Gy/decay	Gy/sec	mGy/day
Electron	1.9E−11	9.1E−09	0.79
Photon	6.6E−13	3.1E−10	0.027

### Morphological abnormalities and total development in silkworm

**Figure 2:**
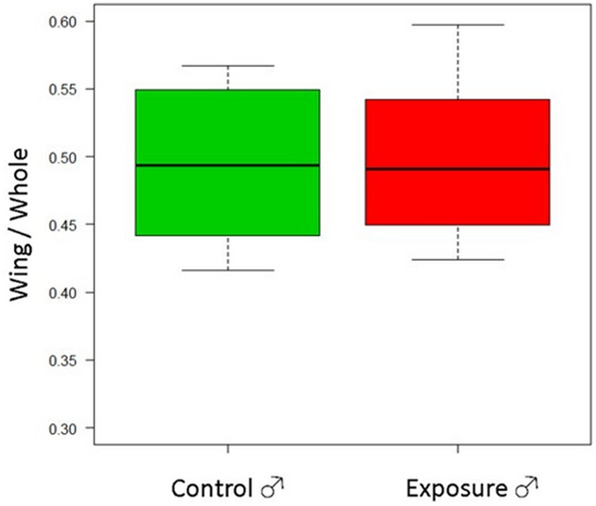
Comparison of the wing-to-whole body length ratio of male silkworm pupae between the exposed and control groups. The number of silkworms in the control and exposed groups was 20 and 30, respectively.

**Figure 3:**
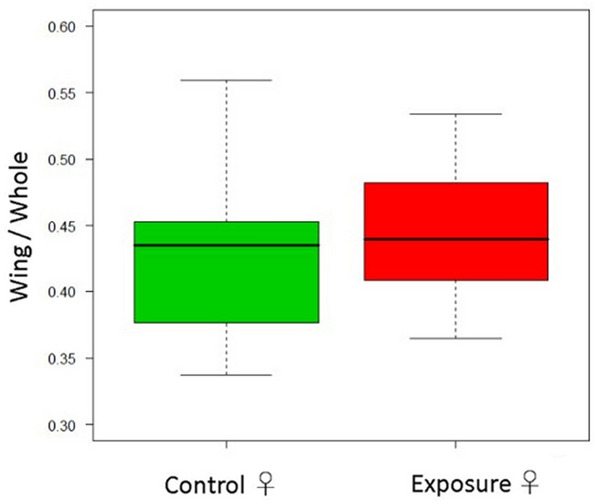
Comparison of the wing-to-whole body length ratio of female silkworm pupae between the exposed and control groups. The number of silkworms in the control and exposed groups were 20 and 30, respectively.

All pupae in the exposed and control groups were fully developed into adults. The wing-to-whole length ratio of silkworm pupae was not significantly different between the exposed and control groups in both males and females (Student *t* test, *p* = 0.76, *p* = 0.16; Figs. 2, 3). The average total absorbed dose by the silkworm was 23 mGy. The average absorbed dose rate of the silkworm was approximately 1.0 mGy/day.

## Discussion

The shrinkage of pupal wing is reportedly a marker of morphological abnormalities caused by external gamma irradiation^30–32^. In this study, the development and wing-to-whole body length ratio of silkworm pupae were compared between the ^137^Cs-exposed and control groups. The results revealed morphological abnormalities in pupal wings that were absent in chronic low-dose exposure with the ingestion of high-concentration ^137^CsCl-supplemented diet. The average absorbed dose rate was higher in internal exposure (0.81 mGy/day) than in external exposure (0.21 mGy/day). The total average absorbed dose-rate was approximately 1.0 mGy/day. To the best of our knowledge, there is no data on the effects of radiation exposure, especially the effects of internal radiation exposure, at such a low dose rate in insects. The estimated dose rate of 1 mGy/day is specified as ‘No information’ in the derived consideration reference levels (DCRLs) of three types of invertebrates, including insects, even with reference to ICRP^1^. Although these results are for domesticated silkworms, the data are essential to estimate the effects of radiation on insects, as the data on the effects of low dose-rate exposure are lacking.

Notably here, the absorbed doses of the silkworm were estimated by directly feeding ^137^CsCl-supplemented artificial feed. The ^137^Cs concentration, 1.3 × 10^3^ Bq/g fresh weight (fw), in the artificial diet can be converted to the ^137^Cs ground deposition of 9 MBq/m^2^, which was relatively high-level contamination area within the Fukushima’s exclusion zone (Fig. 1). Lepidopterans are unlikely to feed on ^137^Cs at such a concentration for a long-term after the FDNPP accident, because internal exposure in lepidopterans mainly results from the ingestion of contaminated food plants. The soil-to-plant transfer factor of Cs in grasses and herb has been estimated to be 6.3 × 10^−2^ and 6.6 × 10^−2^, respectively^33^. Moreover, even if the initial radiocesium transfer factor in herbaceous plants^34^ is considered, except for the effects of direct deposition of radiocesium on leaves, it is unlikely that such plants with a high ^137^Cs concentration can exist in the environment for a long-term. In this experiment, internal exposure upon continued ingestion of such a high concentration of ^137^Cs was simulated; however, the silkworm, a lepidopteran insect, did not show any reduction in wing size in the pupal stage.

In a previous study, a high dose of external gamma irradiation (27 Gy) was required to observe 50% reduction in wing size in the pupae of silkworm, which is considered a radiosensitive strain^31^, suggesting that the silkworm is radioresistant with respect to morphological characteristics. The average absorbed dose for silkworms in the present study was 23 mGy, which is considerably lower than that reported in the previous study. Moreover, in general, the biological effects of radiation are lower under chronic low dose-rate exposure than under high dose-rate exposure, such as that arising from gamma-ray irradiation. These may explain the present findings of no morphological abnormalities in the pupal wing.

Several internal exposure experiments have been conducted in wild lepidopterans such as *Z. maha* and *P. rapae*, which were collected after the Fukushima nuclear accident^20–23^. However, it is difficult to discuss the effects of radiation exposure on these lepidopterans, because the absorbed dose has not been estimated in these previous studies. Besides, quantitative data on the threshold dose for wing size reduction as a morphological abnormality in silkworms using a gamma-ray irradiator have been reported^30–32^. However, as for wild lepidopterans, such data are lacking because studies on the radiation effects in wild lepidopterans have primarily focused on lethality- and sterilization-related doses for pest control, such as in the sterile insect technique^35^. Although it is difficult to evaluate the direct effects of radiation on wild lepidopterans, feeding radiocesium at a concentration considerably lower than that used in this study, reportedly causes morphological abnormalities in wild lepidopteran insects^20–23^. To fill the gap in radio-sensitivity data between silkworms and wild lepidopteran, additional quantitative, direct irradiation experiments in wild lepidopteran insects are warranted.

One of the critical differences between our study and earlier studies is in previous studies, plants contaminated by the transfer of radiocesium from the soil were used to feed lepidopterans^20–23^. In contrast, in our study, the silkworm was fed an artificial diet directly supplemented with ^137^CsCl solution. Some plants are highly radiosensitive, and morphological defects in plants were observed after the FDNPP accident^7,8^. Specific stress-maker genes were expressed in rice plants grown under low-dose radiation exposure on contaminated Chernobyl soil^36^; changes in gene expression in rice plants have also been reported in the contaminated field near the FDNPP^37^. Therefore, when feeding plants collected in a contaminated area or plants that have absorbed radioactive cesium, it may be necessary to consider indirect effects exerted via the forage plants. Otaki and Taira^38^ indicated the possibility of indirect effects of host plants on butterflies, with the plants not being able to synthesize enough thiamine (vitamin B1) following radiation exposure. If nutritional changes occurred in host plants at low-dose radiation, noticeable consequences on the butterfly can be expected. Recently, an internal exposure experiment in the pale grass blue butterfly, *Z. maha,* did not show any morphological abnormalities when *Z. maha* was fed an artificial diet, with a high concentration of ^137^CsCl^39^. This result is in line with the results of this study and negates the direct effects of radiation.

Overall, we have constructed a quantitative experimental system to observe the effect of radiation at low dose-rate exposure on silkworm morphology. To the best of our knowledge, this is the first study to perform an internal exposure experiment on silkworm larvae with an assessment of the absorbed dose. Within the range of our results it is suggest that the morphological abnormalities in silkworm pupae wings could not have occurred by direct radiation exposure to the ^137^CsCl-supplemented diet. In this study, we focused on radiation effects on morphological abnormalities in pupal wings. However, in order to evaluate radiation effects on silkworms more comprehensively, we must evaluate not only the morphology but also reproduction abilities, transgenerational effects, and genetic effects. Targeting silkworm, for which the genome sequence is known, and genetic tools are available, is potentially useful in the assessment for the effects of radiation at a low dose and low dose-rate in future studies. Our quantitative experimental system will provide essential data to evaluate the direct effects of radiation and chronic internal exposure on silkworm. Cumulative data of such fundamental studies will help to assess low dose-rate radiation effects on lepidopterans.

## Materials and methods

### Silkworm strain

The silkworm strain used in this study was NB2 (F1 hybrid xe28 × p20). The silkworms were obtained from the Silkworm Genetic Resource of National Bio-Resource Project at the Kyushu University in Japan.

### Low dose-rate exposure and internal exposure

Radioactive cesium chloride solution (^137^CsCl), which contained 0.05 mg/g CsCl in 0.1 M HCl as a carrier solution was added to the artificial diet of silkworm (Silkmate 2S; Nosan Co., Yokohama, Japan). The concentration was set at 1385 Bq/g fresh weight (fw), which can be converted to ^137^Cs ground deposition of approximately 9 MBq/m^2^ when the radiocesium is distributed within 5 cm from the soil surface and 1.3 g/cm^3^ soil density (Fig. 1)^40^. The contamination level is in the range of initial deposition density of ^137^Cs post-FDNPP accident in the exclusion zone. The silkworms were reared on the contaminated diet from the egg to the fifth instar larval stage, and they had free access to the diet throughout the larval stages. For the control group, a CsCl solution with 0.05 mg/g CsCl in 0.1 M HCl was added to the artificial diet.

### Detection of morphological abnormalities

The wings of silkworm pupae are known to shrink when exposed to radiation^32^. Thus, the wing size is a suitable indicator of morphological abnormalities caused by radiation^31^. In this study, morphological abnormalities were determined by measuring the wing size-to-whole body size ratio of silkworm in the pupal stage (Fig. 1).

### Estimation of the external absorbed dose-rate

**Figure 4:**
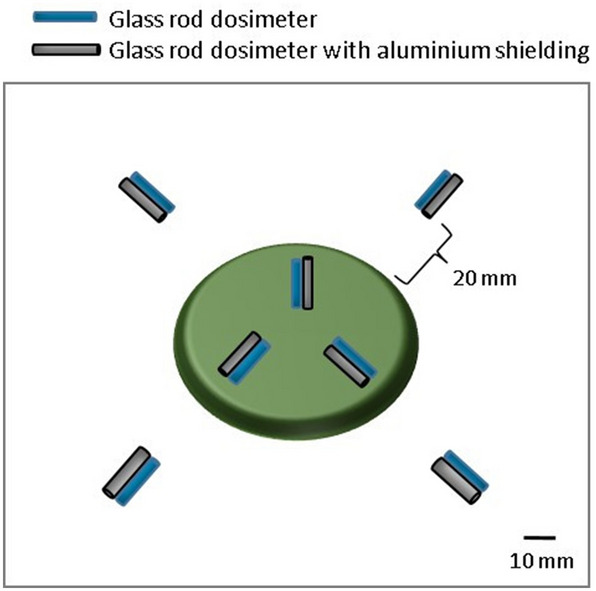
Setting of the glass rod dosimeter on the ^137^CsCl-supplemented artificial diet.

The external absorbed dose rate was estimated using a glass rod dosimeter (diameter 1.5 mm × 12 mm; GD-302 M; AGC Techno Glass Corporation, Shizuoka, Japan) with a reader (FGD-1000; AGC Techno Glass Corporation, Shizuoka, Japan). The beta-rays were separated using approximately 0.6 mm thick aluminium cover. The beta-to-gamma-ray dose ratio was estimated by simple subtraction with and without the aluminium cover. The glass rod dosimeter was placed on the artificial diet (Fig. 4).

### Estimation of the internal dose rate

The internal dose rate was estimated using the Monte Carlo particle transport simulation code with PHITS^41^. The geometry was applied in columnar forms as the shape of silkworm larvae (Supplementary Fig. S1). The density was 1.0 g cm^−3^, and the elements were H (10.1 wt%), C (11.1 wt%), O (76.2 wt%), and N (2.6 wt%), which were composed of the soft tissues defined by the ICRU^42^. The concentration of ^137^Cs in the larvae was determined by gamma spectrometry using a high-purity germanium detector (GEM30-70, ORTEC, USA) with a multi-channel analyzer (Easy-MCA-8k; ORTEC, USA). The ^137^Cs concentration was determined as the average value of five worms, with one measurement per worm (Table 2). Emission data from ^137^Cs were retrieved from the website of National Nuclear Data Center, BNL^43^. Source particles used in the PHITS calculation were 662 keV photon (85.1% per decay) for gamma rays, up to 514 keV (94.7%) and 1176 keV (5.30%) electrons with continuous spectrum for beta rays, 5.84 keV (8.18%, averaged value for two emissions) for Auger electrons, and 630.25 keV (9.57%, averaged value for five emissions) for internal conversion electrons. Low-energy X-rays were not included in the calculations. As we assumed a uniform distribution of ^137^Cs in the worm body, we calculated the absorbed dose with the entire body as one Tally [T-Deposit]. Total internal exposure dose was calculated by adapting the calculated values to the 20-day larval period. This estimation involves uncertainty due to the above assumptions.

### Statistical analyses

The lower (Q1) and upper (Q3) quartiles and the interquartile range (IQR = Q3 − Q1) were calculated for the wing size-to-whole body size ratio. Differences in the ratio were analyzed using the Student’s *t* test. Statistical analyses were performed using R version 3.6.0^44^. The sample size is listed in Supplemental Table S1. The significance level was set at *p* < 0.05 for statistical procedures.

## Supplementary information

Supplementary information.

## Data Availability

Almost all data are included in this article and Supplementary Information files. The data sets not included in this article and Supplementary Information files are available from the corresponding author upon reasonable request.
