# Author Correction: Observation of morphological abnormalities in silkworm pupae after feeding ^137^CsCl-supplemented diet to evaluate the effects of low dose-rate exposure

**DOI:** 10.1038/s41598-021-85664-x

**Published:** 2021-03-22

**Authors:** Sota Tanaka, Tadatoshi Kinouchi, Tsuguru Fujii, Tetsuji Imanaka, Tomoyuki Takahashi, Satoshi Fukutani, Daisuke Maki, Akihiro Nohtomi, Sentaro Takahashi

**Affiliations:** 1 Research Group for Environmental Science, Japan Atomic Energy Agency, Tokai, Ibaraki 319-1195 Japan; 2 Division of Radiation Life Science, Institute for Integrated Radiation and Nuclear Science, Kyoto University, Kumatori-cho, Sennan-gun, Osaka, 590-0494 Japan; 3 Laboratory of Creative Science for Insect Industries, Graduate School of Bioresource and Bioenvironmental Sciences, Kyushu University, Nishi-ku, Motooka, Fukuoka 819-0395 Japan; 4 Division of Nuclear Engineering Science, Institute for Integrated Radiation and Nuclear Science, Kyoto University, Kumatori-cho, Sennan-gun, Osaka, 590-0494 Japan; 5 Technical Staff Office, Institute for Integrated Radiation and Nuclear Science, Kyoto University, Kumatori-cho, Sennan-gun, Osaka, 590-0494 Japan; 6 Quantum Radiation Sciences, Department of Health Sciences, Graduate School of Medical Sciences, Kyushu University, Maidashi, Higashi-ku, Fukuoka City, 812-8582 Japan; 7 Professor Emeritus, Kyoto University, Kitashirakawa Oiwake-cho, Sakyo-ku, Kyoto, 606-8502 Japan

Correction to: Scientific Reports 10.1038/s41598-020-72882-y, published online 29 September 2020.

This Article contains errors in the Discussion section.

“The ^137^Cs concentration, 1.3 × 10^3^ Bq/g fresh weight (fw), in the artificial diet can be converted to the ^137^Cs ground deposition of 9 MBq/m^2^, which was relatively high-level contamination area within the Fukushima’s exclusion zone (Fig. 1).”

should read:

“The ^137^Cs concentration, 1.3 × 10^3^ Bq/g fresh weight (fw), in the artificial diet can be converted to the ^137^Cs ground deposition of 90 MBq/m^2^, which was higher than high-level contamination area within the Fukushima’s exclusion zone (Fig. 1).”

In the Materials and methods section under subheading ‘Low dose-rate exposure and internal exposure’.

“The concentration was set at 1385 Bq/g fresh weight (fw), which can be converted to ^137^Cs ground deposition of approximately 9 MBq/m^2^ when the radiocesium is distributed within 5 cm from the soil surface and 1.3 g/cm^3^ soil density (Fig. 1)^40^.”

should read:

“The concentration was set at 1385 Bq/g fresh weight (fw), which can be converted to ^137^Cs ground deposition of approximately 90 MBq/m^2^ when the radiocesium is distributed within 5 cm from the soil surface and 1.3 g/cm^3^ soil density (Fig. 1)^40^.”

