# Supplementary material for: Observation of morphological abnormalities in silkworm pupae after feeding 137CsCl-supplemented diet to evaluate the effects of low dose-rate exposure
Source: Sci Rep. 2020 Sep 29;10:16055. doi: 10.1038/s41598-020-72882-y (PMC7524783; doi:10.1038/s41598-020-72882-y)
Supplement: Supplementary file 1 — Supplementary information. [file 41598_2020_72882_MOESM1_ESM.pdf]

Scientific Reports

Supplementary Information

Observation of morphological abnormalities in silkworm pupae after feeding ^{137}CsCl-supplemented diet to evaluate the effects of low dose-rate exposure

Sota Tanaka[1*], Tadatoshi Kinouchi[2], Tsuguru Fujii[3], Tetsuji Imanaka[4], Tomoyuki Takahashi[4], Satoshi Fukutani[4], Daisuke Maki[5], Akihiro Nohtomi[6], Sentaro Takahashi[7]

[1] Research Group for Environmental Science, Japan Atomic Energy Agency, Tokai, Ibaraki 319-1195, Japan

[2] Division of Radiation Life Science, Institute for Integrated Radiation and Nuclear Science, Kyoto University, Kumatori-cho, Sennan-gun, Osaka 590-0494, Japan

[3] Laboratory of Creative Science for Insect Industries, Graduate School of Bioresource and Bioenvironmental Sciences, Kyushu University, Nishi-ku, Motooka, Fukuoka, 819-0395, Japan

[4] Division of Nuclear Engineering Science, Institute for Integrated Radiation and Nuclear Science, Kyoto University, Kumatori-cho, Sennan-gun, Osaka 590-0494, Japan

[5] Technical Staff Office, Institute for Integrated Radiation and Nuclear Science, Kyoto University, Kumatori-cho, Sennan-gun, Osaka 590-0494, Japan

[6] Quantum Radiation Sciences, Department of Health Sciences, Graduate School of Medical Sciences, Kyushu University, Maidashi, Higashi-ku, Fukuoka City, 812-8582 Japan

[7] Professor emeritus, Kyoto University, Kitashirakawa Oiwake-cho, Sakyo-ku, Kyoto 606-8502, Japan

* Corresponding author E-mail address: tanaka.sota@jaea.go.jp (S. Tanaka)

Table S1 Wing and whole-body length of silkworm pupae in control group

Control♂				Control♀			
No.	Wing (mm)	Whole body (mm)	wing/whole	No.	Wing (mm)	Whole body (mm)	wing/whole
1	10.60	24.08	0.44	1	10.37	25.77	0.40
2	11.49	20.80	0.55	2	12.45	26.08	0.48
3	10.07	22.63	0.44	3	10.70	24.58	0.44
4	10.21	23.34	0.44	4	8.20	24.30	0.34
5	11.09	20.80	0.53	5	8.70	24.26	0.36
6	12.17	22.18	0.55	6	11.58	24.41	0.47
7	10.10	21.39	0.47	7	10.34	23.45	0.44
8	10.89	22.11	0.49	8	9.93	24.30	0.41
9	11.29	20.53	0.55	9	9.81	26.87	0.36
10	10.03	21.26	0.47	10	10.26	23.06	0.45
11	10.85	20.99	0.52	11	10.84	24.52	0.44
12	9.71	22.15	0.44	12	9.30	23.32	0.40
13	10.31	20.83	0.49	13	9.34	25.54	0.37
14	11.64	20.54	0.57	14	9.46	25.11	0.38
15	11.12	20.60	0.54	15	11.97	21.40	0.56
16	8.95	21.48	0.42	16	11.30	24.56	0.46
17	9.09	20.52	0.44	17	11.17	23.22	0.48
18	9.47	22.02	0.43	18	10.88	24.47	0.44
19	11.35	20.15	0.56	19	9.65	25.62	0.38
20	10.88	19.54	0.56	20	11.32	25.98	0.44

Table S2 Wing and whole-body length of silkworm pupae in $^{137}CsCl$-supplemented group

Cs-137♂				Cs-137♀			
No.	Wing (mm)	Whole body (mm)	wing/whole	No.	Wing (mm)	Whole body (mm)	wing/whole
1	9.08	20.18	0.45	1	9.65	22.62	0.43
2	10.68	19.35	0.55	2	9.41	19.85	0.47
3	10.13	20.64	0.49	3	9.59	21.39	0.45
4	10.36	19.64	0.53	4	9.82	24.17	0.41
5	8.60	18.42	0.47	5	10.13	27.76	0.36
6	9.34	19.04	0.49	6	10.09	27.01	0.37
7	10.06	18.57	0.54	7	10.45	24.03	0.43
8	11.23	20.26	0.55	8	9.90	19.50	0.51
9	9.88	19.04	0.52	9	10.31	19.31	0.53
10	9.04	20.26	0.45	10	9.59	18.80	0.51
11	10.05	21.33	0.47	11	9.18	22.41	0.41
12	9.82	18.12	0.54	12	10.42	20.84	0.50
13	8.70	20.52	0.42	13	9.81	22.26	0.44
14	10.10	20.83	0.49	14	10.45	21.53	0.49
15	9.47	21.52	0.44	15	9.02	23.79	0.38
16	9.98	21.51	0.46	16	9.88	21.31	0.46
17	10.31	18.87	0.55	17	10.67	21.10	0.51
18	10.01	17.45	0.57	18	8.90	21.05	0.42
19	11.36	19.08	0.60	19	9.48	21.61	0.44
20	9.08	20.27	0.45	20	9.76	22.15	0.44
21	9.52	21.83	0.44	21	10.39	26.86	0.39
22	9.35	21.86	0.43	22	10.08	20.91	0.48
23	9.30	18.98	0.49	23	10.00	21.35	0.47
24	9.64	22.57	0.43	24	9.48	23.54	0.40
25	10.93	21.07	0.52	25	9.47	20.39	0.46
26	10.06	19.79	0.51	26	9.72	22.27	0.44
27	10.33	17.30	0.60	27	9.35	22.33	0.42
28	10.13	20.74	0.49	28	11.01	21.63	0.51
29	10.18	18.53	0.55	29	9.40	24.05	0.39
30	10.03	19.04	0.53	30	9.95	24.36	0.41

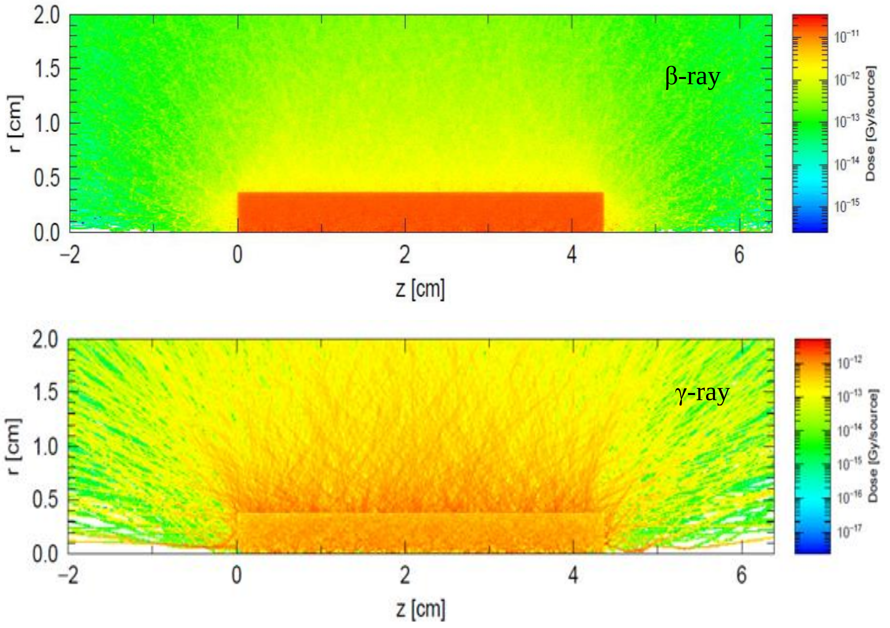

Fig. S1 Behavior of radiation from ^{137}Cs in silkworm larvae simulated by PHITS
